# New azoxystrobin clay carrier to control corn late wilt disease

**DOI:** 10.1007/s11274-026-04876-3

**Published:** 2026-03-14

**Authors:** Ariel Hadad, Elhanan Dimant, Peleg Hadari, Eden Etedgi, Giora Rytwo, Ofir Degani

**Affiliations:** 1 Migal – Galilee Research Institute, Tarshish 2, Kiryat Shmona, 1101600 Israel; 2 Faculty of Sciences, Tel-Hai University of Kiryat Shmona and the Galilee, Upper Galilee, Tel-Hai, 1220800 Israel

**Keywords:** Bentonite, *Cephalosporium maydis*, Crop protection, *Harpophora maydis*, Pathogenicity, Real-time PCR, Sepiolite

## Abstract

Corn late wilt disease (LWD), caused by the fungus *Magnaporthiopsis maydis*, poses a significant threat to corn production in highly impacted regions. Its increasing global relevance is exacerbated by climate change, which facilitates the pathogen’s spread and prevalence. This research supports efforts to broaden LWD management beyond resistant varieties, whose effectiveness can fail within years under high disease pressure. In this study, a novel approach involving the slow release of azoxystrobin from clay carriers was assessed. Two growth-room experiments demonstrated that at the disease’s latent stage (up to 20 days), seed treatment with a clay-azoxystrobin (clay-AS) formulation had minimal impact on early growth indices. However, by day 40, the sepiolite-based treatment increased plant shoot weight by 61%, although pathogen infection levels, monitored via qPCR, remained elevated. A subsequent full-season potted trial revealed significant benefits from applying sepiolite-AS and bentonite-AS formulations directly to the seedbed. At 42 days post-planting, these treatments markedly enhanced plant survival (191% for sepiolite-AS and 64% for bentonite-AS) and improved phenological development (175% and 67%, respectively) compared to unprotected controls, alongside a notable 95% reduction in root infection. By harvest (78 days), bentonite-AS emerged as particularly effective, increasing shoot biomass by 128% and ear yield by 135%. Bentonite-AS treatment reduced cob and shoot symptoms by 23% and 42%, respectively, and nearly eradicated pathogen levels. This research demonstrates that clay-based fungicide formulations effectively mitigate LWD impacts. The method reduces fungicide use and offers a potentially scalable alternative, is adaptable across diverse agricultural systems, and shows potential applicability in managing other soil-borne diseases.

**Supplementary Information:**

The online version contains supplementary material available at 10.1007/s11274-026-04876-3.

## Introduction

Corn (maize, *Zea mays*) is the leading world grain crop (Tanklevska et al. 2020; Zhao et al. 2017), together with rice and wheat (Food and Agriculture Organization of the United Nations. (2023). FAOSTAT: Food and Agriculture Commodity Production data. Retrieved from http://www.fao.org/faostat/en/#data/QC). It is considered a staple food for humans and fodder for animals. In addition, it has applications in various industries, including producing food additives, oils, starch, paper, and biofuel (Tanklevska et al. 2020). However, the full yield potential of corn crops is often not reached due to a range of diseases. Late wilt disease (LWD) caused by the fungus *Magnaporthiopsis maydis* (former names *Harpophora maydis* and *Cephalosporium maydis*) is considered a major threat to commercial corn production in Israel (Drori et al. 2013) and Egypt (Agag et al. 2021; Kamara et al. 2021). These two countries are considered the world’s most challenged areas by LWD (Degani 2021), probably because of their preferable climatic conditions and Egypt’s long history of LWD prevalence (Ghazy et al. 2024). Additionally, the disease is considered severe in Spain (Ortiz-Bustos et al. 2019), Portugal (Ortiz-Bustos et al. 2015), and India (Sunitha et al. 2021).

The pathogen infects corn plants early in the growing season, colonizes the vascular tissue, obstructs water transport to the upper plant parts, and leads to wilting and collapse approximately two weeks before harvest (Abdelghany et al. 2025; Matos et al. 2024). In Israel, the disease has been prevalent for approximately 40 years, primarily in the northern Upper Galilee, particularly in the Hula Valley (Degani 2022). Over this period, the disease has intensified and expanded, with mortality rates approaching 100% in some fields planted with susceptible cultivars. (Degani et al. 2019b). Such cases depend on the level of soil infestation, which plays a significant role in the disease development (Yassin and El-Naggar 2024). The fungus impairs seed germination and root development. Initial symptoms of dehydration typically appear approximately 50 days after sowing (DAS), starting at the lower parts of the plant and progressing upward. Symptoms include leaf and lower stem yellowing and wilting, yellow-brown discoloration of vascular bundles, and cob damage. Seeds, if produced, are underdeveloped, and infected seeds may serve as sources for disease spread (Matos et al. 2024).

The pathogen association with other plant pathogenic fungi like *Fusarium verticillioides*, the causal agent behind stalk rot, and *Macrophomina phaseolina*, the charcoal rot disease agent (all grouped in the post-flowering stalk rot disease complex), can enhance the damage to the maize plants (Degani et al. 2020; Elsayed et al. 2023; Khokhar et al. 2014; Shofman and Degani 2025a). *Magnaporthiopsis maydis* survives in the soil and on corn plant residues for extended periods. It may also persist, typically without obvious symptoms or with only mild disease signs, on alternative host plants such as lupine (Sahab et al. 1985), cotton, watermelon, and *Setaria viridis* (Dor and Degani 2019). These secondary hosts facilitate the pathogen’s survival even when crop rotation practices are employed.

Current LWD management primarily involves an integrated agrotechnical approach based on principles of avoidance, exclusion, and eradication (Gordani et al. 2023). Over the years, various control strategies have been evaluated, including agrotechnical practices (such as balanced fertilization and soil flooding, no till, cover crops, crop rotation, and minimizing soil disturbance), nanoparticle additives, chemical control, biological methods, physical treatments (solar disinfection), and plant-derived compounds (Degani 2022; Matos et al. 2024). Despite the promising results obtained with some of these strategies, the sole method currently employed in many regions to effectively manage the disease remains the cultivation of resistant corn varieties (Abdelghany et al. 2025).

While chemical control of diseases is becoming increasingly problematic due to its implications on the environment and health safety, and subsequent tightening supervision over their use, it is still the most effective means to manage LWD and other diseases in severe cases (Degani et al. 2025c; Zhou et al. 2025). Such an approach was established over the years, for example (El-Moghazy et al. 2017; Singh and Siradhana 1989). From 2015 to 2018, an efficient and economical chemical LWD control method was developed in Israel (Degani et al. 2018, 2019b, 2020). The method is based on controlled application of the pesticide azoxystrobin via drip irrigation at three intervals (15, 30, and 45 DAS). Adjusting the cultivation method to paired-row planting significantly reduced associated costs. In heavily infected fields with disease-susceptible plants, this treatment completely eliminated the pathogen’s DNA in the roots and stalks, reduced disease symptom occurrence by 41%, and enhanced overall crop yield and quality by 36% and 77%, respectively, matching levels typically observed in healthy fields. Additionally, combining azoxystrobin with formulations employing alternative mechanisms of action demonstrated effectiveness and could lower the risk of pathogen resistance development.

However, the adjustments in cultivation practices and drip irrigation systems are not universally applicable and involve high costs. Along with the public concerns regarding chemical uses, the potential for pathogens evolving resistance to such compounds (and in particular to Qo-inhibiting fungicide azoxystrobin) (Avila-Adame and Koller 2003; Corkley et al. 2022) remains a serious problem. Consequently, significant research efforts are currently directed toward developing environmentally friendly alternatives for managing corn late wilt disease. Studies conducted in recent years have demonstrated promising results using the biocontrol fungus *Trichoderma* spp. against the LWD pathogen (*M. maydis*) (Elmeihy et al. 2025; Elshahawy and El-Sayed 2018). In Israel, several species within this genus underwent rigorous evaluations, starting from laboratory tests and controlled growing room trials, culminating in comprehensive field experiments spanning an entire growing season. These biocontrol agents significantly improved plant growth and yield indices, reaching levels comparable to healthy plants, and reduced pathogen concentrations in plant tissues by up to 98% (Degani and Dor 2021; Degani et al. 2021a, b). However, despite these benefits, the effectiveness of biological treatments remains dependent on environmental conditions and can be limited in cases of severe disease pressure (Ons et al. 2020). Consequently, combining low-dose azoxystrobin with biological treatments has been investigated to stabilize and enhance the efficacy of biocontrol against LWD while reducing the risk of fungicide resistance (Gordani et al. 2023; Matos et al. 2025).

Here, we propose a novel approach involving the slow release of azoxystrobin from selected clay carriers, designed to eliminate the need for drip irrigation or modifications to cultivation practices. This method provides localized protection to sprouts during their vulnerable early growth stage, when fungal penetration and establishment typically occur. In recent decades, clay-based substrates have been extensively investigated to mitigate the ecological impact of pesticide use by reducing rinsing, degradation, and loss of activity (Aranda et al. 2018; Mishael et al. 2003; Rytwo and Rabinowitz 2012; Rytwo et al. 2015; Sheng et al. 2001; Shuali et al. 2011; Undabeytia et al. 2011). Clay is an inexpensive and widely available material with no known environmental toxicity concerns. Numerous studies have demonstrated the potential of various clays to serve as carriers for the slow release of active compounds, protecting them from leaching, evaporation, and photodegradation (Nir et al. 2013). These clays can be applied in their natural state or modified with organic cations to optimize adsorption, release dynamics, and bioactivity. A recent example is the successful use of clay for the slow release of essential oils to control thrips in net house-grown chives, without phytotoxic effects (Shaltiel-Harpaz et al. 2023).

Despite these advantages, clay-based carriers for pesticide delivery in the context of LWD management have received limited attention. Formulations based on clay—applicable via seed coating or strip placement at sowing—offer a cost-effective and environmentally friendly alternative to conventional chemical treatments. Such approaches reduce pesticide inputs and can be implemented under diverse field conditions without requiring specialized infrastructure. The slow release of azoxystrobin from clay particles placed in the sowing strip enables prolonged protection of corn seedlings during their vulnerable growth stages while minimizing fungicide loss through soil movement. Moreover, this localized application strategy supports the integration of chemical and biological control methods and allows for a significant reduction in pesticide usage. Importantly, clay-based azoxystrobin (clay-AS) formulations may also confer protection against additional soilborne pathogens, as demonstrated for *Macrophomina phaseolina* (Degani et al. 2024).

The present study develops and evaluates clay-based substrates (sepiolite and bentonite) for the adsorption and sustained release of azoxystrobin (Degani et al. 2026). Bentonite is a platelet-structured clay primarily composed of montmorillonite, while sepiolite is a naturally occurring fibrous clay mineral. While raw bentonite is not considered a good sorbent for non-cationic organic molecules, sepiolite is well known for its high capacity to adsorb organic compounds (Shuali et al. 2011). Their efficacy against the *M. maydis* was tested in both early-stage sprouts (under controlled growth room conditions) and full-season plants under net house conditions. Disease suppression was assessed through plant growth and yield measurements, as well as molecular tracking of pathogen DNA in plant roots using quantitative real-time PCR (qPCR).

## Materials and methods

### Rationale and research design

The study comprised three experiments with similar designs. The first two experiments (conducted in 2023 and 2025) focused on growth-room corn sprouts grown under controlled conditions for up to 40 days. The third was conducted in pots placed within a net house, over a full growth season lasting up to 78 days. Using pots offers several advantages over traditional open-field trials, including enhanced uniformity of environmental conditions and precise control over inoculum pressure, irrigation, soil composition, and fertilization. This controlled setup also minimizes cross-contamination between treatments through randomized pot distribution and enables the inclusion of mock controls—uninfected, healthy plants serving as a baseline for comparison. Two application methods of the novel clay-azoxystrobin (clay-AS) formulations were evaluated: (1) seed coating and (2) direct addition of the formulation to the sowing pit (seedbed application). The formulations were designed to model low-dose, sustained fungistatic activity (Degani et al. 2026) rather than maximal fungicide exposure. The seed coating approach was implemented only in the 2023 growth room experiment. This method was excluded from the subsequent experiments due to the challenges in achieving uniform and precise coating with the dry powder, along with observed adverse effects on seed health.

### The fungi used in this study

The *Magnaporthiopsis maydis* isolate *Mm2* (CBS 133165, Westerdijk Fungal Biodiversity Institute, Utrecht, The Netherlands) was obtained from diseased sweet corn (maize, *Zea mays* cv. Jubilee) plants collected in commercial fields in Sde Nehemia, Upper Galilee, northern Israel (Drori et al. 2013). This isolate has been previously characterized based on pathogenicity and physiological traits, colony morphology, microscopic features, and molecular identification (Shofman et al. 2022). The colony and microscopic characteristics of *Mm2* closely resemble those of *M. maydis* strains described in Egypt and India (Payak et al. 1970; Samra et al. 1963). The aggressiveness of the *Mm2* isolate was previously evaluated in full-season potted corn plants under net house conditions and classified as moderately virulent (Shofman and Degani 2025b). This moderately virulent, well-characterized Mm2 isolate was selected to reflect typical field conditions and ensure comparability with previous studies.

### Growth of the fungi

*Magnaporthiopsis maydis* colonies were cultured on potato dextrose agar (PDA; Difco, Detroit, MI, USA) in 90 mm Petri dishes and incubated in the dark at 28 ± 1 °C for 4–5 days. For subculturing, a 6 mm agar disk was excised from the actively growing margin of a 5–7-day-old colony and transferred to the center of a fresh PDA plate, which was then incubated under the same conditions.

### Azoxystrobin clay-based formulation preparation

**Fig. 1:**
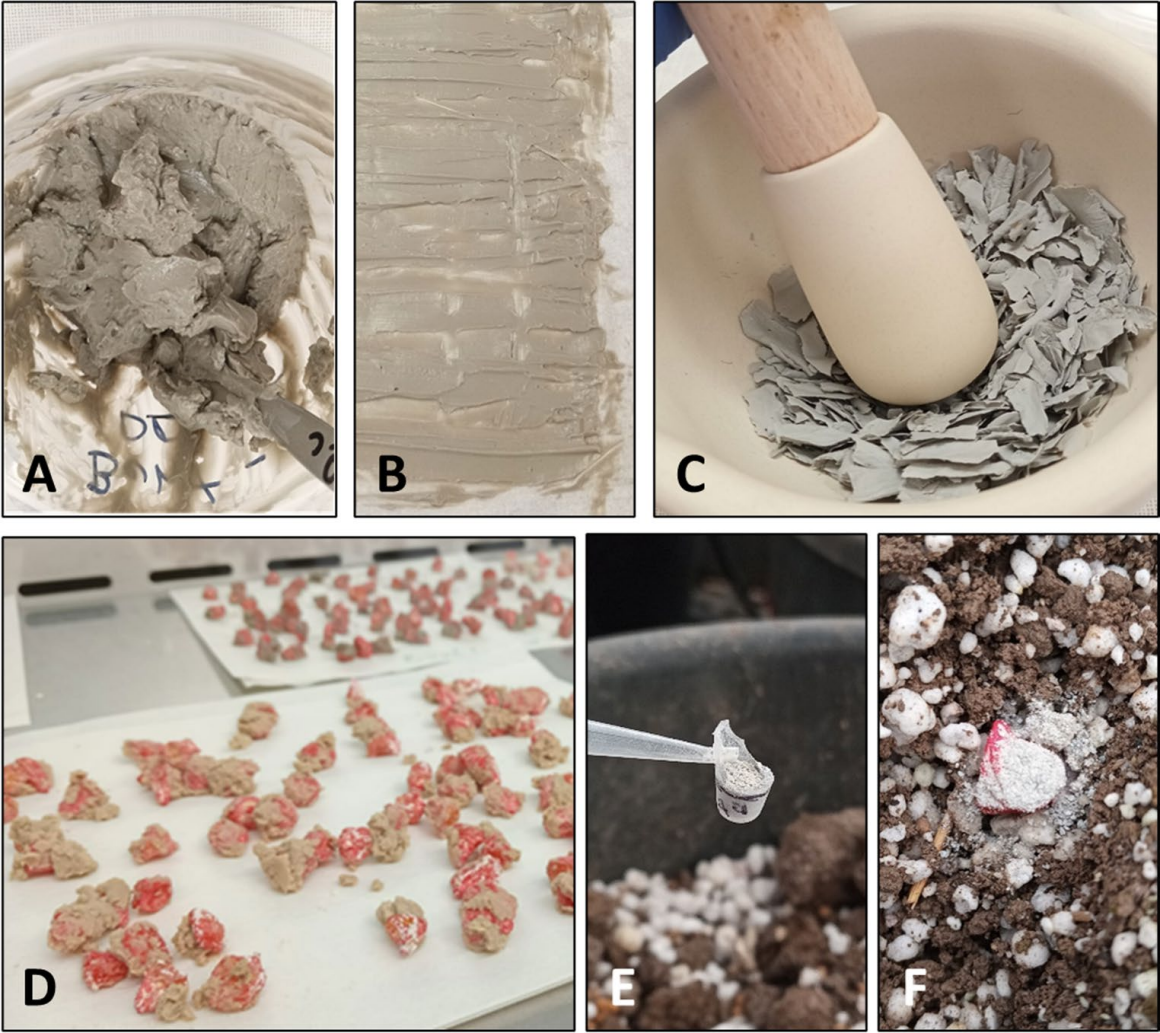
Preparation and application of clay-azoxystrobin (clay-AS) formulations using sepiolite and bentonite as carriers. **A**,** B** Homogenization and spreading the clay–AS mixture on paper for drying; **C** grinding the dried formulation into a fine powder; **D** application via seed coating; **E**,** F** application by adding 60 ± 5 g clay (in formulation with azoxystrobin or as sole ingredient) to each seed

In this study, two types of clay minerals were used—bentonite (CAS No: 1302-78-9) and sepiolite (CAS No: 63800-37-3)—both purchased from Sigma–Aldrich (Rehovot, Israel). For the formulation, a commercial azoxystrobin-based fungicide (Amistar S.C., 25% w/v; Syngenta, Basel, Switzerland; supplied by Adama Makhteshim, Ashdod, Israel) was used. A volume of 76.9 mL of a tenfold-diluted commercial azoxystrobin solution was thoroughly mixed with 23.1 g of clay on baking paper to form a homogeneous slurry. As a control, the same volume of deionized distilled water (DDW) was used in place of azoxystrobin. The tenfold dilution of the commercial azoxystrobin formulation (Amistar S.C.) was selected as a practical experimental choice to enable accurate handling and homogeneous incorporation into clay carriers. The selected dose was aligned with a previously published clay–Az study on cotton charcoal rot, in which this formulation effectively suppressed pathogen infection without inducing consistent phytotoxic effects (Degani et al. 2026). The mixtures were left to dry at room temperature for up to two days, until the complete evaporation of water. The resulting dry materials (ca. 23–28 g) were then ground into fine powders using a mortar and pestle and applied via seed coating or by adding the formulation onto the seedbed (Fig. 1).

### Inoculation method

In all three experiments, the inoculation protocol included pre-sowing soil inoculation using sterilized *M. maydis*–infected millet (*Panicum miliaceum*) grains, followed by complementary infection using fungal colony agar disks (Sect.  2.3). Inoculum quantities were adjusted according to pot volume (30 g in 2.5-L pots in the growth-room experiments; 100 g in 10-L pots in the full-season trial) to achieve comparable disease pressure under growth-room and net-house conditions, based on prior validated pot assays (Degani et al. 2023, 2024; Gordani et al. 2023). Soil inoculation was carried out by incorporating the infected millet grains into the upper soil layer (~ 5 cm depth) one week prior to sowing. To maintain consistent pathogen pressure, two additional inoculations were applied approximately one and two weeks after sowing, each consisting of the addition of 15 *M. maydis* colony agar disks per pot.

#### Preparation of infected sterilized millet grains

Millet seeds were pre-infected with *M. maydis* following the method of Gordani et al. (Gordani et al. 2023), with slight modifications. One kilogram of dry millet grains was soaked for 1 h in boiling tap water, drained, and transferred into a 2 L glass jar. Gypsum powder (CaSO₄·2 H₂O; 13 g per jar) was added and thoroughly mixed with the grains to adjust pH and prevent clumping. The grains were sterilized in an autoclave at 120 °C for 60 min. An 8-day-old *M. maydis* colony grown on PDA in a 9 cm-diameter Petri plate (including the agar medium) was cut into small pieces and aseptically added to the jar. The contents were mixed with a sterile spatula to evenly distribute the inoculum. Jars were loosely sealed to allow gas exchange, covered with aluminum foil, and incubated at 28 ± 1 °C in darkness for 7–14 days, until the mycelium fully colonized the grains.

### Growth room sprouts’ experiment

#### Experimental design

The growth room experiment was conducted in two independent biological replicates (conducted in 2023 and 2025). The first experiment lasted 20 days and evaluated both seed coating and seedbed application methods. The second experiment focused exclusively on the seedbed method and extended to 40 days. Total of 11 treatments included inoculation with *M. maydis* (strain *Mm2*), either alone or in combination with bentonite or sepiolite clays—applied with or without azoxystrobin. An azoxystrobin irrigation treatment (positive control) and a non-infected (healthy) control group were included for comparison.

Each of the 11 treatments and controls (in the two independent experiments) consisted of nine biological replicates (pots). Due to late wilt disease mortality, the final number of surviving repeats is indicated for each figure. Plants were grown in 2.5 L plastic pots (upper diameter: 16 cm; height: 17 cm; lower diameter: 12 cm), filled with 2.5 L of non-sterilized local field soil (North R&D plantation farm, Hula Valley, Upper Galilee, northern Israel, 33°09′08.2″ N 35°37′21.6″ E), mixed in a volume-to-volume ratio with 33% Perlite No. 4 for improved aeration. The field soil was selected to closely replicate actual cultivation conditions. This soil had no documented history of *M. maydis* infestation, and any potential prior contamination was assumed to be minimal. Soil fertilization in the 2023 experiment was carried out by incorporating 10 g of Osmocote 14-4-28 (ScottsMiracle-Gro, Marysville, OH, USA), a controlled-release fertilizer with a four-month nutrient release period, into the upper 5 cm of soil in each pot, in accordance with the manufacturer’s instructions. In the 2025 trial, Osmocote Pro 8–9 (18-9-10) was used instead, as its higher nitrogen and phosphorus contents are slightly better suited to supporting 40-day maize seedling growth.

Five seeds of corn cultivar ‘Prelude’ (SRS Snowy River Seeds, Australia; distributed by Green 2000 Ltd., Israel) were sown in each pot at a depth of ca. 3 cm. According to the supplier and prior experience, this cultivar typically reaches maturity in 79 days and produces cobs measuring approximately 18.9 cm in length and 5.3 cm in diameter (Shofman and Degani 2025b). The seeds were commercially pre-treated with a standard pesticide mixture containing Thiram, Captan, Carboxin, and Metalaxyl-M. Prior to sowing, seed samples were tested to confirm high viability. Plants were grown under controlled conditions in a growth room set to a 16 h light / 8 h dark photoperiod (automated lighting), with temperature maintained at 25–28 °C and relative humidity at 45–65%. Irrigation was applied every two days (4 times a week), typically with 66.7 mL of tap water per pot. The watering volume was adjusted throughout the growth period to meet the plants’ needs. Phenological development was monitored using the corn staging system described by Abendroth et al. (Abendroth et al. 2011). Thinning was carried out on day 20 (V3-4 stage), leaving one uniform and representative seedling per pot until the conclusion of the experiment at day 40 (V5-7 stage), corresponding to the onset of the plant’s vegetative growth phase.

#### Protective treatments

 For the seed-coating procedure, each clay type was placed separately in large mixing bowls. Corn seeds designated for sowing were lightly moistened with water, then added to the bowls and gently shaken to ensure uniform coating with clay from all directions. The coated seeds were subsequently transferred onto clean baking paper and allowed to dry completely. In the growth room sprout experiment using the seedbed method, 50 ± 5 g of sepiolite and 60 ± 5 g of bentonite (per seed), either formulated with azoxystrobin or applied alone, were added at the time of sowing. As a positive control, azoxystrobin irrigation was carried out on the day of sowing, 9 days after sowing (DAS), and 18 DAS. At each irrigation event, 10 mL of Amistar S.C. solution (1.25 mL of the commercial product, corresponding to 0.3125 g of active ingredient) was applied to each pot, while control pots received an equal volume of tap water. Across the three irrigation events, the cumulative dose was 0.9375 g active ingredient per pot. The solution was delivered using a pipette directly into the drip stream of the designated pots, and irrigation continued for 1 min and 42 s. This procedure was performed exclusively on the scheduled irrigation days to prevent the treatments receiving fungicide from experiencing excess or insufficient watering.

#### Assessment of plant growth and health

Plant development and LWD severity were assessed at two time points: at thinning (20 DAS) and at the end of the experiment (40 DAS). The following parameters were recorded: plant survival rate, plant height, fresh weight of above-ground parts, and phenological stage (leaf count). In addition, root tissue samples were collected from all plants at both time points and stored at − 20 °C for subsequent DNA extraction and molecular analysis.

### Net house full-season pots trial

#### Growth conditions

The experiment was conducted in 10-L pots placed in a net house at the Avnei Eitan Experimental Farm in the Golan Heights, north-eastern Israel (32° 49′ 03.3″ N 35° 45′ 46.4″ E). Pots were filled with local peat soil originating from the farm with no known history of late wilt disease; any background infection was therefore considered negligible. To improve aeration, the soil was amended with 33% perlite (v/v).

Five seeds of maize cv. Prelude, originating from the same batch used in the growth-room experiment, were sown per pot on 10 June 2024. Seedling emergence was assessed at 7 and 14 DAS, and thinning to one plant per pot was performed at 42 DAS. Each treatment included nine biological replicates, with the pot/plant serving as the experimental unit. Final harvest was conducted at 78 DAS; the surviving n for each treatment and time point is reported in the figure captions.

Irrigation was supplied via a computerized drip system, with daily volumes increased from 667 mL to 1333 mL per pot during the growing season to accommodate plant development. Irrigation management and applications against non-LWD pathogens were performed as required, following the Ministry of Agriculture’s recommended corn cultivation protocol. During the experimental period, the average temperature was 26.9 °C (minimum 14.1 °C, maximum 39.8 °C), and the average relative humidity was 65.3% (minimum 15.0%, maximum 96.0%), conditions previously reported to favor late wilt disease development (Degani et al. 2021b; Singh and Siradhana 1987a).

#### Protective treatments

In the whole-season net-house trial, the protective treatments with both clays, sepiolite and bentonite, were applied at a rate of 60 ± 5 g per seed. Azoxystrobin irrigation control treatment was not included in this trial for practical reasons, primarily to maintain a relatively high number of replications within the available experimental space. The application rates and treatment set reflect a proof-of-concept pot system rather than field-scale recommendations.

#### Assessment of plants’ growth and health

Plant growth parameters were recorded at the thinning stage and at the end of the experiment. These included: survival rate, plant height, fresh weight of the above-ground parts, phenological stage (number of leaves), and flower count. Root samples were also collected and immediately stored at − 20 °C for subsequent DNA extraction and purification. At the end of the trial (78 DAS), additional parameters were assessed, including cob weight and the severity of late wilt symptoms on the cob spathes (the leafy bracts surrounding the developing ear). Wilt symptoms were visually categorized into four severity levels: (1) completely dried, (2) severe (clearly visible wilting symptoms), (3) mild (minor dehydration signs), and (4) healthy (Fig. 1) (Degani et al. 2021b).

### Real-time PCR (qPCR) *M. maydis* DNA analysis

Quantitative PCR (qPCR) was employed to quantify *M. maydis* DNA in maize root tissues (Degani and Gordani 2022). Unless otherwise stated, samples were collected from nine plants per treatment group. Plant tissues were thoroughly rinsed with running tap water, surface sterilized by sequential immersion in 1% sodium hypochlorite (NaOCl) and sterile distilled water for 10 min each, then cut into ~ 2 cm segments. For each biological replicate, 0.7 g of tissue was used.

Total DNA (plant and fungal) was extracted using a modified CTAB protocol, as previously described (Degani et al. 2019a; Murray and Thompson 1980). DNA was isolated from plant tissue, purified by chloroform–isoamyl alcohol extraction, precipitated with isopropanol, resuspended in ultra-pure water, and stored at − 20 °C until qPCR analysis. Detection of *M. maydis* was carried out using a SYBR Green-based qPCR assay optimized with species-specific primers (A200) targeting a unique genomic region identified via amplified fragment length polymorphism (AFLP) analysis (Saleh and Leslie 2004; Zeller et al. 2000). To normalize *M. maydis* DNA levels, the mitochondrial cytochrome c oxidase (COX) gene served as a housekeeping reference (Almquist 2016; Baskarathevan et al. 2016; Garrido et al. 2009), using the COX F/R primer pair (Table 1). Relative quantification was performed using the ΔCt method, assuming equal amplification efficiency across samples. The COX primers, initially designed for dicots, were previously validated for maize (Degani et al. 2019b). Product specificity and amplification efficiency were confirmed by melt curve analysis and amplification plots.

**Table 1:** Primers for *Magnaporthiopsis maydis* detection. ^1^

Pairs	Primer	Sequence	Uses	Amplification	References
Pair 1	A200a-for A200a-rev	*5′-CCGACGCCTAAAATACAGGA-3′* *5′-GGGCTTTTTAGGGCCTTTTT-3′*	Target gene	200 bp *M. maydis* species-specific fragment	(Drori et al. 2013)
Pair 2	COX-F COX-R	*5′-GTATGCCACGTCGCATTCCAGA-3′* *5′-CAACTACGGATATATAAGRRCCRR AACTG-3′*	Control	Mitochondrial Cytochrome C oxidase (*COX*) gene product	(Li et al. 2006; Weller et al. 2000)

^1^ The R symbol signifies Guanine or Adenine (purine). The synthesized primer included a mixture of primers with both nucleotides

Amplifications were carried out using the CFX384 Real-Time PCR Detection System (Bio-Rad, Hercules, CA, USA) on 384-well plates. The qPCR reaction composition is in Table 2. Thermal cycling conditions included an initial activation step at 95 °C for 60 s, followed by 40 cycles of denaturation at 95 °C for 15 s and annealing/extension at 60 °C for 30 s. A melting curve analysis was performed at the end of the run to verify amplification specificity.

**Table 2:** Composition of the qPCR reaction mixture (total volume 5 µl) ^1^

Component	Volume per reaction (µl)	Final concentration/description
Forward primer	0.25	10 µM
Reverse primer	0.25	10 µM
iTaq™ Universal SYBR Green Supermix ^2^	2.5	1×
DNA template	2.0	Diluted DNA

^1^ All qPCR reactions were performed with four technical replicates per sample. ^2^ Bio-Rad Laboratories Ltd., Hercules, CA, USA

### Statistical analysis

Experiments followed a completely randomized design. For each outcome, we defined the experimental unit as one pot (thinned at mid-season to a single plant), and all analyses used the pot-level value. Data analysis was performed using GraphPad Prism software (version 10.5.0 (774), GraphPad Software Inc., San Diego, CA, USA), dated 27/05/2025. Normality of the data distribution was assessed using the Shapiro–Wilk test. When the data met the assumption of normality (*p* > 0.05), a one-way analysis of variance (ANOVA) was conducted, followed by Fisher’s least significant difference (LSD) test at a significance threshold of *p* < 0.05. For datasets that did not meet the normality assumption (*p* < 0.05), the nonparametric Kruskal–Wallis test was applied, followed by Dunn’s multiple comparisons test (uncorrected). Due to the inherent challenges in achieving uniform pathogen inoculation across all pots, a high degree of variability in the results, especially in the net house experiment, was expected. This variability, as indicated by elevated standard error values, may have reduced the ability to detect statistically significant differences between treatments.

## Results

**Fig. 2:**
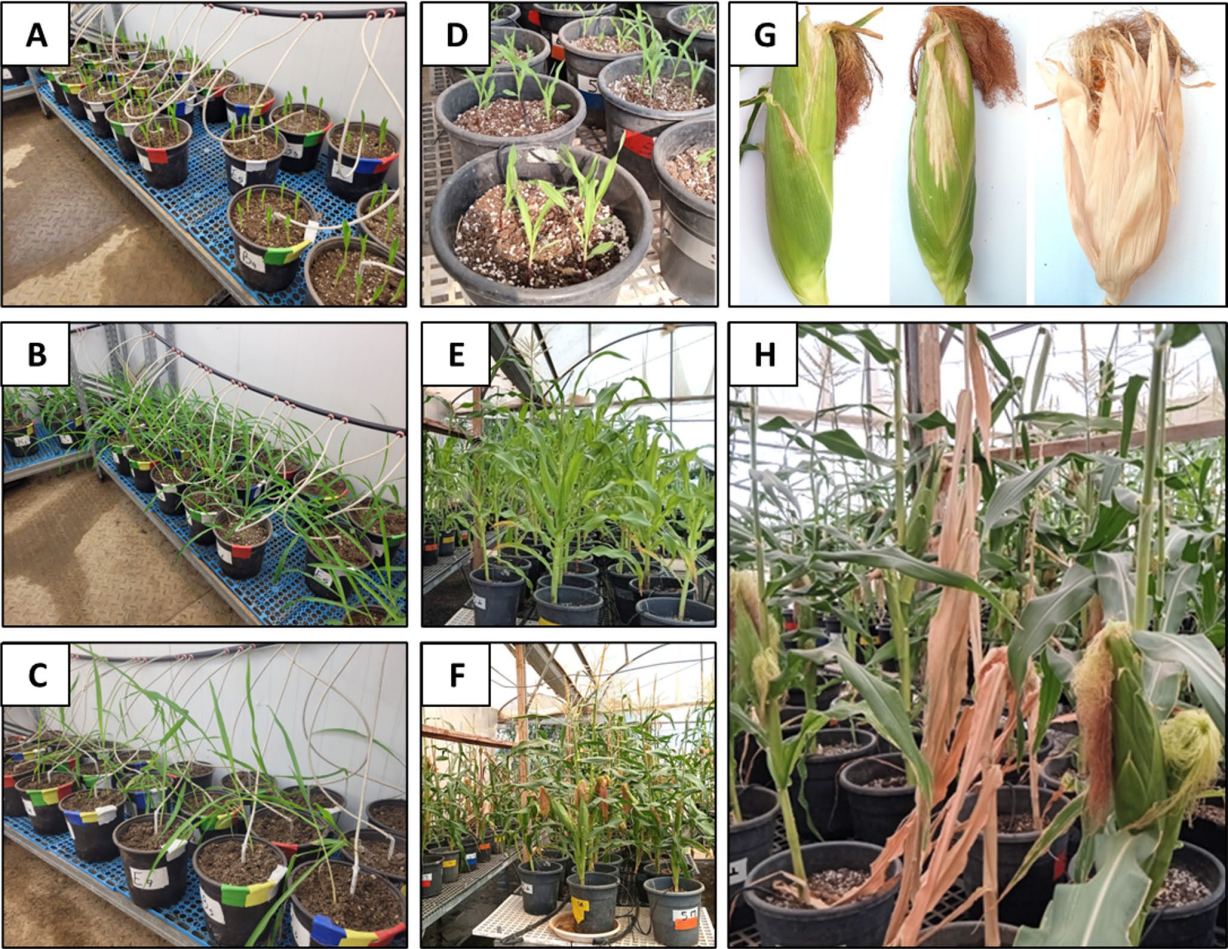
Pot experiments assessing the effect of bentonite and sepiolite clay formulations adsorbed with azoxystrobin on late wilt disease in corn. Treatments were applied either as seed coatings or by direct addition to the sowing pit. **A**–**C** Controlled conditioned growth room images taken 14 **A**, 20 **B**, and 40 **C** days after sowing. **D**–**H** Net-house images taken 9 **D**, 42 **E**, and 79 **F**–**H** days after sowing. **G** Cobs’ spathes showing late wilt symptoms of minor (left), moderate (middle), and severe (right) intensity. **H** Severely diseased and wilted corn plant, 79 days after sowing

In this study, the protective potential of a clay-azoxystrobin (Amistar S.C., clay-AS) powder formulation against the early stages of late wilt disease was evaluated. The efficacy of selected treatments was examined in two growth room sprout experiments (conducted in 2023 and 2025) and a full-season net-house pot trial, yielding promising results across all experimental setups, as detailed below. Bentonite and sepiolite clays were tested as carriers for the fungicide in pot experiments, with a clay-water-treated group serving as the control (Fig. 1). The efficacy of the formulation was assessed through both seed treatment and seedbed application in a sweet corn variety highly susceptible to late wilt disease (Fig. 2).

### The growth room sprouts 2023 experiment

In the 2023 growth room experiment, the addition of the formulations directly to the sowing pit (seedbed application) was evaluated at the latent stage of the disease (20 days after sowing, DAS). At this age, plants exhibited comparable growth across all treatments, as indicated by similar shoot biomass, height, and phenological development (Fig. S1). Although molecular detection of the pathogen did not yield statistically significant differences among the infected groups, the sepiolite-AS treatment resulted in a 50% reduction in pathogen presence. Conversely, the bentonite-AS treatment—and to a greater extent, bentonite alone—led to elevated infection levels, with increases of 37% and 200%, respectively.

In comparison, seed coating with the clay-AS formulations under pathogen stress caused a non-significant 12–15% decrease in shoot biomass (Fig. S2). Nevertheless, the sepiolite-AS application achieved a sharp 92% suppression of root infection, while the corresponding bentonite treatment was ineffective, showing a 76% increase in infection. Furthermore, seed coating with either clay alone (without fungicide) resulted in a pronounced increase in infection levels—exceeding 600% relative to the infected control without clay treatment. These results are most likely attributed to the clay coating, which impairs water uptake during germination. Consequently, and given the technical challenge of achieving a precise and uniform seed coating, in the 2025 growth room repeat and full-season net house trials, the clay formulations were applied directly into the sowing hole.

### The growth room sprouts 2025 experiment

**Fig. 3:**
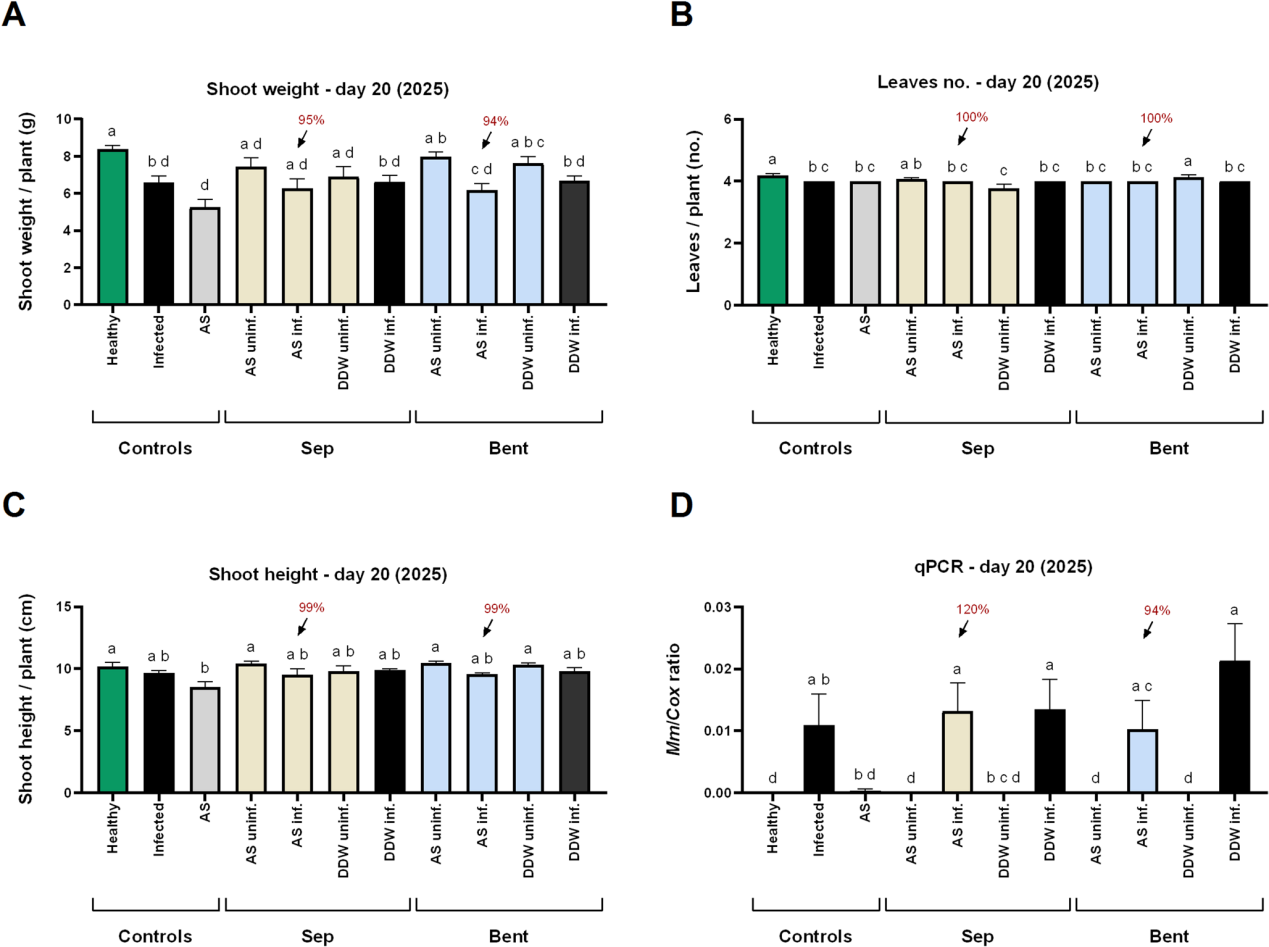
Growth and pathogen infection parameters in the 2025 growth room pot experiment, assessed 20 days after sowing. Treatments consisted of azoxystrobin (Amistar S.C.; Syngenta, Basel, Switzerland) adsorbed onto sepiolite and bentonite clays (yellow and blue bars, respectively), applied directly to the sowing pit at sowing. **A** shoot fresh weight, **B** phenological development (leaf count), **C** shoot height, and **D** relative quantity of *M. maydis* (*Mm*) DNA in root tissues, normalized to plant cytochrome c oxidase (*Cox*) gene levels. Controls: Healthy (green bars) or uninf. – non-inoculated; Infected or DDW inf. (black bars) – inoculated without AS-based treatments; AS irrigation (gray bars) – Amistar S.C. (1.25 mL commercial product) applied via irrigation on days 0, 9, and 18 after sowing. Abbreviations: Sep – sepiolite; Bent – bentonite; AS – azoxystrobin; DDW – clay mixed with deionized distilled water (no fungicide). Bars represent the mean of 6–9 biological replicates; error bars indicate standard error. Different letters (a–d) above bars indicate statistically significant differences between treatments (p < 0.05), based on one-way ANOVA **A** or Kruskal–Wallis test **B**–**D**

In the 2025 repeat’s seedbed application, similar plant growth outcomes were observed across treatments (Figs. 3A–C). Similarly, pathogen levels in the roots, quantified by qPCR, revealed no significant differences among treatments at this stage (Fig. 3D).

**Fig. 4:**
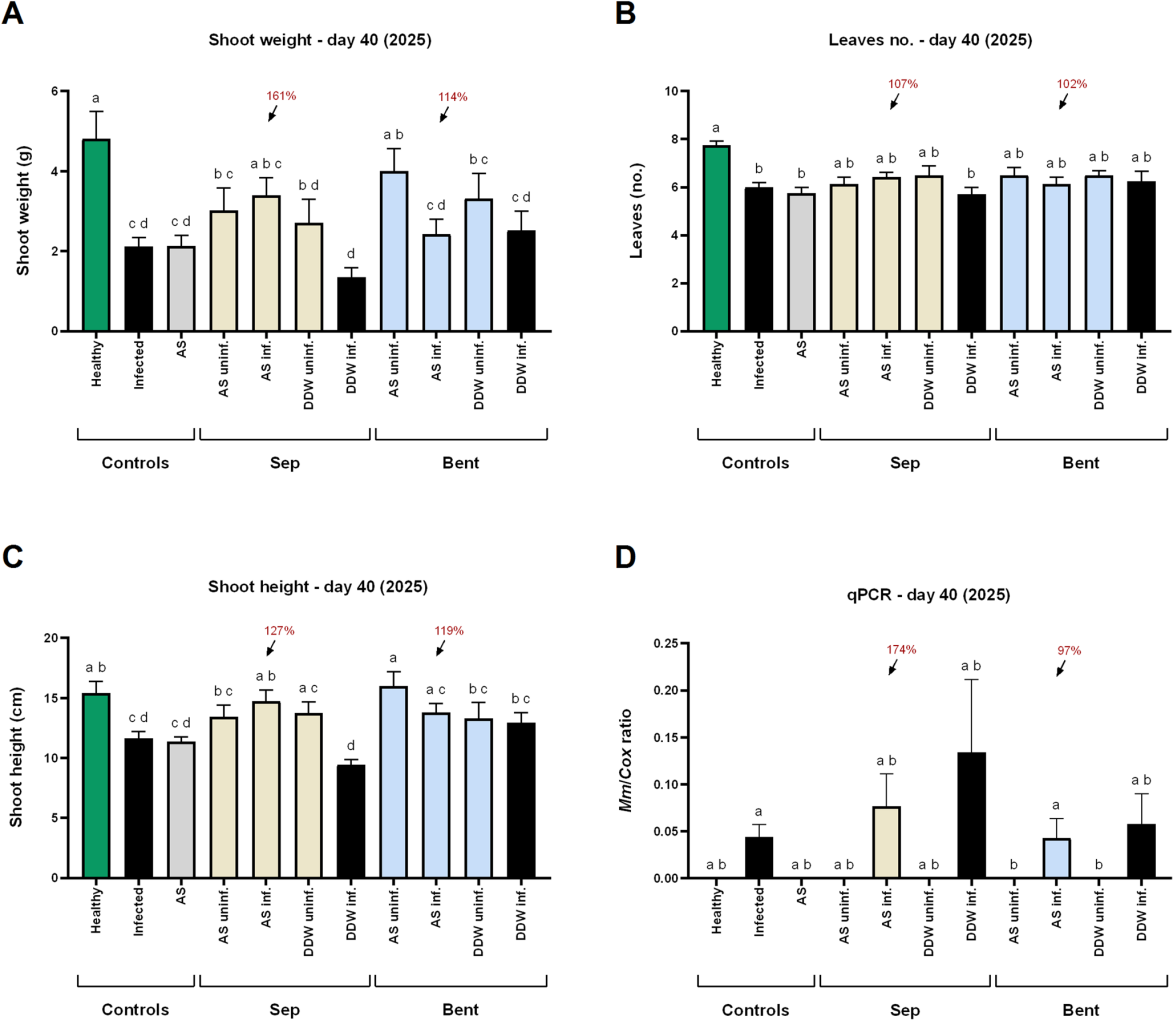
Effect of clay–azoxystrobin formulations on corn plant growth and M. maydis infection in the 2025 growth room experiment, assessed 40 days after sowing. Treatments consisted of azoxystrobin adsorbed onto sepiolite or bentonite clays (yellow and blue bars, respectively), applied directly to the sowing pit at sowing. **A** shoot fresh weight, **B** phenological development (leaf number), **C **shoot height, and **D** relative quantity of *M. maydis* (*Mm*) DNA in root tissues, normalized to plant cytochrome c oxidase (*Cox*) gene levels. Controls: Healthy (green bars) or uninf. – non-inoculated; Infected or DDW inf. (black bars) – inoculated without AS-based treatments; AS irrigation (gray bars) – Amistar S.C. (1.25 mL commercial product) applied via irrigation on days 0, 9, and 18 after sowing. Abbreviations: Sep – sepiolite; Bent – bentonite; AS – azoxystrobin; DDW – clay mixed with deionized distilled water (no fungicide). Data represent the mean ± standard error of 6–8 biological replicates. Different letters (a–d) above bars indicate statistically significant differences between treatments (*p* < 0.05), based on one-way ANOVA **A**, **C** or Kruskal–Wallis test **B**, **D**

By the end of the sprouting phase, approaching the typical onset of LWD symptoms (~ 50 DAS), the beneficial effects of the clay-AS treatments—particularly the sepiolite-AS formulation—began to manifest in the growth parameters (Figs. 4A–C). Statistically significant differences form the infected control were measured in plant height. Application of sepiolite-AS and bentonite-AS enhanced shoot biomass by 61% and 14%, respectively, and increased shoot height by 27% (*p* < 0.05) and 19%. These treatments had a minimal influence on phenological development. At this stage, LWD symptoms were limited (without significant differences among treatments), with only 1–3 out of 9 plants per treatment showing minor signs such as root rot and plant mortality. Despite the initial improvements in plant growth, *M. maydis* root infection levels remained statistically similar to the infected control (Fig. 4D), with notable increases of 74% and 206% following sepiolite-AS and bentonite-alone applications, respectively.

### Net house, full-season pots trial

**Fig. 5:**
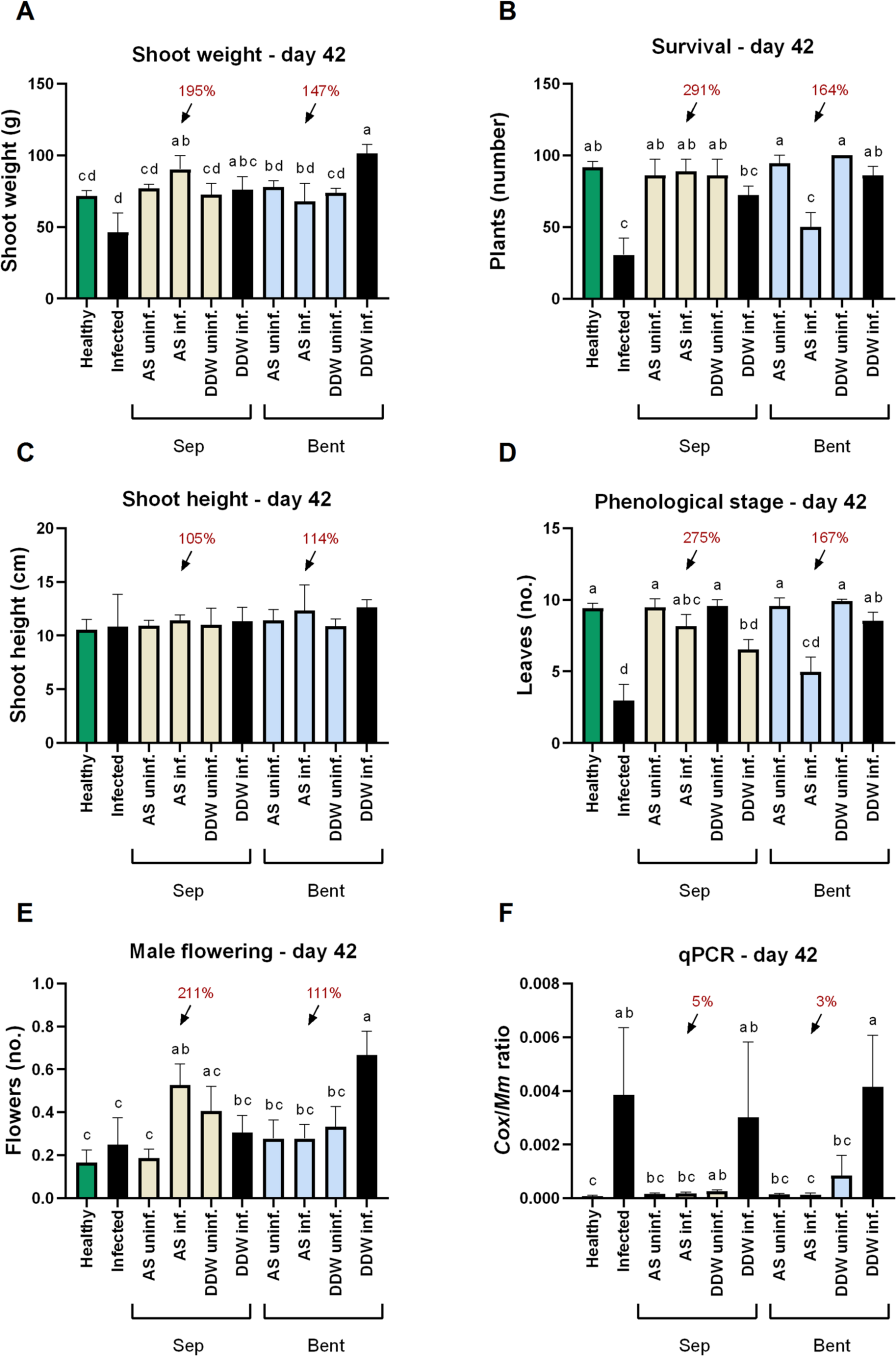
Effect of clay–azoxystrobin formulations on late wilt disease in corn grown in the net house, assessed 42 days after sowing. Treatments included azoxystrobin adsorbed onto sepiolite or bentonite clays (yellow and blue bars, respectively), applied directly to the sowing pit at sowing. **A** shoot fresh weight, **B** survival rate, **C** shoot height, **D** phenological development (leaf number), **E** male flowers count, and **F** relative quantity of *M. maydis* (*Mm*) DNA in root tissues, normalized to plant cytochrome c oxidase (*Cox*) gene levels. Controls: Healthy (green bars) or uninf. – non-inoculated; Infected or DDW inf. (black bars) – inoculated without AS-based treatments. Abbreviations: Sep – sepiolite; Bent – bentonite; AS – azoxystrobin; DDW – clay mixed with deionized distilled water (no fungicide). Data represent the mean ± standard error of 8–9 biological replicates. Different letters (a–d) above bars indicate statistically significant differences between treatments (*p* < 0.05), based on one-way ANOVA **C** or Kruskal–Wallis test **A**, **B**, **D**–**F**

At the above-surface emergence assessment conducted 7 DAS, the application of the sepiolite-AS formulation or bentonite alone to the seedbed significantly (*p* < 0.05) improved plant performance compared with the infected, untreated control (Table S1). A similar trend was observed in the survival assessment performed on day 42 (Fig. 5).

At the mid-season assessment (day 42), the sepiolite-AS treatment corroborated the growth room findings, demonstrating the highest efficacy in protecting plants against late wilt disease. This treatment resulted in significant (*p* < 0.05) improvements in survival (191%), shoot biomass (95%), phenological stage (175%), and male flowering (111%) compared with the infected, untreated control. Moreover, qPCR analysis revealed a substantial 95% (*p* < 0.05) reduction in pathogen infection following the sepiolite-AS seedbed application. In comparison, the bentonite-AS treatment produced a non-significant 47–67% increase in shoot growth (with only an 11% increase in male flowering) but still achieved high pathogen suppression, reducing infection levels by 97% (*p* < 0.05). Notably, in the qPCR molecular analysis, very low threshold cycle (Ct) readings were detected in the uninfected plants grown in control farm soil, likely reflecting a minimal background presence of *M. maydis* in that soil.

**Fig. 6:**
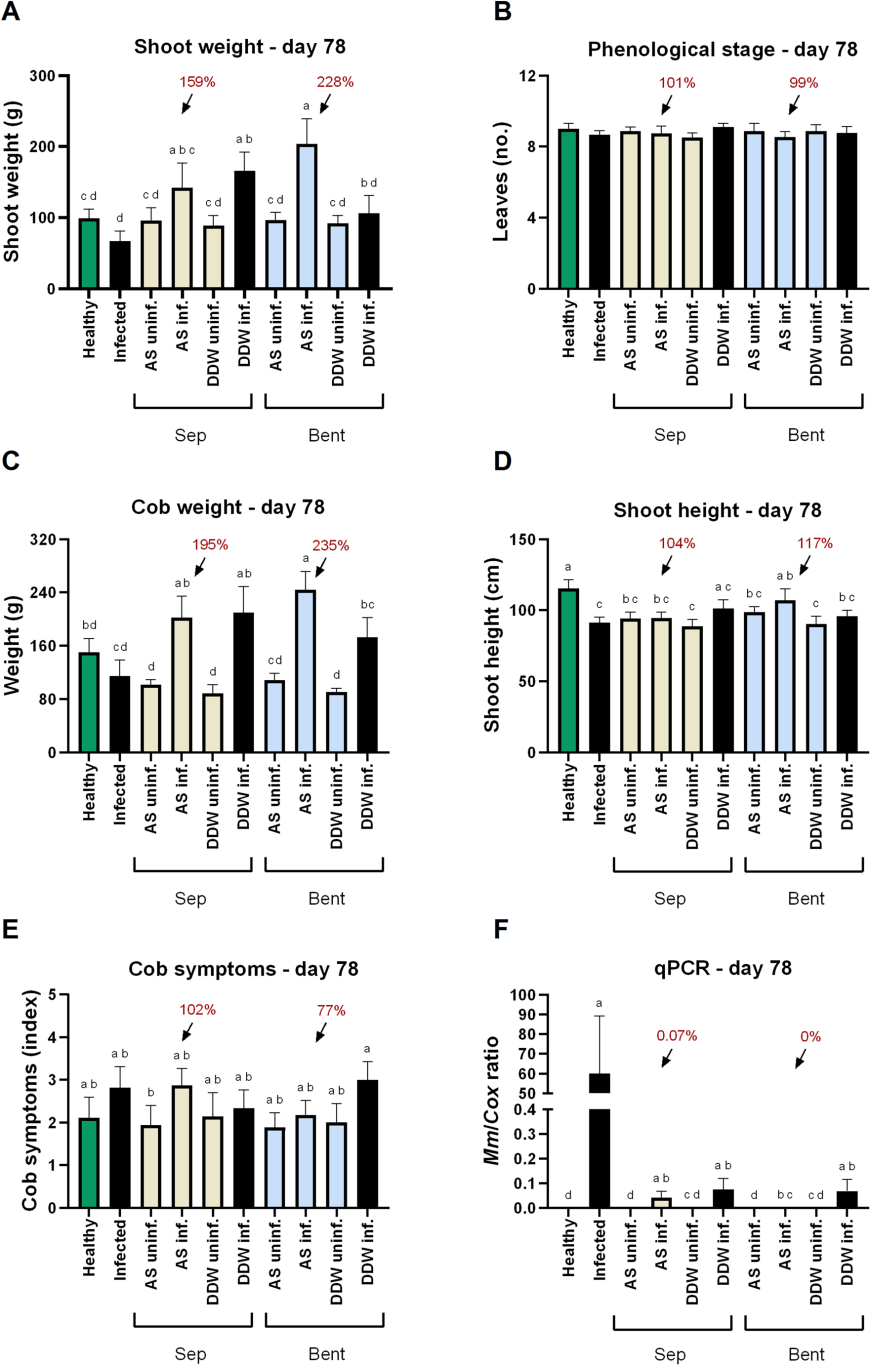
Effect of clay–azoxystrobin formulations on late wilt disease in corn grown in the net house, assessed 78 days after sowing. Treatments included azoxystrobin adsorbed onto sepiolite or bentonite clays (yellow and blue bars, respectively), applied directly to the sowing pit at sowing. **A** shoot fresh weight, **B** phenological development (leaf number), **C** cob weight, **D** shoot height, **E** cob spathes symptoms, and **F** relative quantity of *M. maydis* (*Mm*) DNA in root tissues, normalized to plant cytochrome c oxidase (*Cox*) gene levels. Controls: Healthy (green bars) or uninf. – non-inoculated; Infected or DDW inf. (black bars) – inoculated without AS-based treatments. Abbreviations: Sep – sepiolite; Bent – bentonite; AS – azoxystrobin; DDW – clay mixed with deionized distilled water (no fungicide). Data represent the mean ± standard error of 7–9 biological replicates due to outlier subtraction (using the ROUT method in GraphPad Prism software, with the Q = 1% stringency setting, as recommended by the software guidelines). Different letters (a–d) above the bars indicate statistically significant differences between treatments (*p* < 0.05), determined using one-way ANOVA **A**, **C** or the Kruskal–Wallis test **B**, **D**–**F**

By harvest (growth day 78), corresponding to the acute disease stress stage, the sepiolite-AS formulation maintained its high efficacy, providing significant (*p* < 0.05) improvements in shoot biomass (59%) and cob weight (95%) compared with the infected, untreated control (Fig. 6). This treatment also reduced root pathogen levels to near zero. At this stage, the full potential of the bentonite-AS treatment became evident. While it had conferred only mild protection against *M. maydis* by the end of the sprouting stage, it proved most effective during the disease acute burst phase, resulting in significant (*p* < 0.05) increases in shoot fresh biomass (128%), shoot height (17%), and cob weight (135%). Most notably, the bentonite-AS application achieved near-complete pathogen suppression (*p* < 0.05; Fig. 6).

Comparative analysis of the total impact (relative to the infected, untreated control) across the two growth room experiments, and the net house trial demonstrates the overall beneficial effect of the clay-based treatments (Table 3). On growth day 20, growth differences among treatments were modest, reaching up to ca. 25%. However, by day 40, these differences increased markedly to approximately 90%. At this stage, the sepiolite-AS formulation under late wilt stress achieved an average growth improvement of 85%, matching the performance of the healthy, uninfected mock control. In contrast, the bentonite-AS treatment produced only 30% improvement on day 40. Nevertheless, by the end of the season (day 78), bentonite–AS–treated plants reached their peak performance, showing a 70% growth enhancement and a 32% reduction in disease symptoms—comparable to the healthy control plants.

**Table 3:** A comparative evaluation of the growth and health indices

A.														
Treatment		2023 growth room day 20 (average/pot)					2025 growth room day 20 (average/pot)					Total growth (day 20)		
		Survival (%)	Weight (g)	Height (cm)	Leaves (no.)	qPCR	Survival (%)	Weight (g)	Height (cm)	Leaves (no.)	qPCR	Average	Rank	
Controls	Healthy	100%	119%	105%	101%	1%	100%	119%	105%	105%	1%	107%	4	
	AS	98%	80%	89%	95%	36%	97%	80%	89%	100%	4%	91%	10	
Sepiolite	AS uninf.	90%	94%	105%	104%	5%	100%	113%	108%	102%	0%	102%	8	
	**AS inf.**	**96%**	**86%**	**98%**	**107%**	**50%**	**94%**	**95%**	**99%**	**100%**	**120%**	**97%**	**9**	
	DDW uninf.	97%	124%	118%	115%	57%	97%	105%	102%	94%	1%	106%	6	
	DDW Inf.	92%	122%	114%	122%	41%	100%	101%	102%	100%	123%	107%	5	
Bentonite	AS uninf.	93%	125%	122%	116%	6%	100%	121%	109%	100%	0%	111%	3	
	**AS inf.**	**93%**	**120%**	**121%**	**112%**	**137%**	**100%**	**94%**	**99%**	**100%**	**94%**	**105%**	**7**	
	DDW uninf.	93%	148%	127%	120%	20%	100%	116%	107%	103%	1%	114%	1	
	DDW Inf.	100%	161%	123%	125%	300%	100%	101%	102%	100%	195%	114%	2	
B.														
Treatment		2025 growth-room day 40 (average/pot)				2025 net house day 42 (average/pot)						Total growth (ca. day 40)		
		Weight (g)	Height (cm)	Leaves (no.)	qPCR	Survival (%)	Weight (g)	Height (cm)	Leaves (no.)	Male flowers (no.)	qPCR	Average	Rank	
Controls	Healthy	228%	133%	129%	0%	300%	155%	97%	317%	67%	2%	181%	5	
	AS	101%	98%	96%	0%	-	-	-	-	-	-	98%	10	
Sepiolite	AS uninf.	143%	116%	102%	0%	282%	166%	101%	319%	75%	4%	168%	7	
	**AS inf.**	**161%**	**127%**	**107%**	**174%**	**291%**	**195%**	**105%**	**275%**	**211%**	**5%**	**185%**	**2**	
	DDW uninf.	128%	118%	108%	0%	282%	157%	102%	322%	163%	7%	176%	6	
	DDW Inf.	64%	81%	95%	306%	236%	164%	105%	220%	122%	78%	140%	8	
Bentonite	AS uninf.	190%	138%	108%	0%	309%	168%	106%	321%	111%	4%	184%	3	
	**AS inf.**	**114%**	**119%**	**102%**	**97%**	**164%**	**147%**	**114%**	**167%**	**111%**	**3%**	**130%**	**9**	
	DDW uninf.	157%	111%	108%	0%	327%	160%	101%	334%	133%	22%	183%	4	
	DDW Inf.	119%	115%	104%	131%	282%	219%	117%	287%	267%	108%	189%	1	
C.														
Treatment		2025 net house day 78 (plant/pot)							Total growth (day 78)		Total health (day 78)		Total all experiments	
		Weight (g)	Height (cm)	Leaves (no.)	Cob weight (g)	Cob health (index)	Shoot health (index)	qPCR	Average	Rank	Average	Rank	Average	Rank
Co.	Healthy	219%	126%	104%	247%	125%	120%	0%	174%	1	122%	5	160%	1
Sepiolite	AS uninf.	107%	103%	102%	97%	131%	130%	0%	103%	7	131%	4	147%	4
	**AS inf.**	**159%**	**104%**	**101%**	**195%**	**98%**	**115%**	**0.07%**	**140%**	**4**	**106%**	**8**	**130%**	**7**
	DDW uninf.	99%	97%	98%	85%	124%	115%	0%	95%	9	119%	6	143%	5
	DDW Inf.	186%	111%	105%	202%	117%	120%	0.13%	151%	3	119%	7	117%	8
Bentonite	AS uninf.	108%	108%	103%	104%	133%	142%	0%	106%	6	138%	1	155%	2
	**AS inf.**	**228%**	**117%**	**99%**	**235%**	**123%**	**142%**	**0%**	**170%**	**2**	**132%**	**3**	**135%**	**6**
	DDW uninf.	103%	99%	103%	87%	129%	138%	0%	98%	8	133%	2	150%	3
	DDW Inf.	119%	105%	101%	166%	93%	98%	0.11%	123%	5	96%	9	113%	9

A. Summary of the growth room pot trials – growth and survival indices from the two experiments, measured on day 20 after sowing. Values are expressed as % difference vs. infected untreated control at the same time point: %Δ = 100 × (Value Treatment /Value Infected Control). For qPCR, lower values indicate reduced infection and are converted to % reduction accordingly. A total average impact was then calculated for each treatment, and treatments were ranked according to this average (from highest impact – rank 1, to lowest – rank 9 or 10). The sepiolite and bentonite AS formulations are indicated in bold

B. Summary of the growth room and full-season net house pot trials – growth and survival indices measured on day 40 (growth room) or day 42 (net house). The sepiolite and bentonite AS formulations are indicated in bold

C. Summary of the growth and health indices from the end of the net house trial (day 78), followed by an overall summary of all experiments and the combined total effect of each treatment. The sepiolite and bentonite AS formulations are indicated in bold

## Discussion

*Magnaporthiopsis maydis*, the causal agent of late wilt disease (LWD), is an emerging phytopathogen that affects corn plants in highly impacted areas (Matos et al. 2024). In Israel, it poses a major threat to corn production, often leading to plant death in the late growth stages, with few (but often not practical or costly) effective control measures currently available (Degani 2022). Despite extensive efforts and the evaluation of various control strategies over the years, effective management of LWD relies mainly on resistant cultivars (Abdelghany et al. 2025), which require constant effort to develop new hybrids since highly pathogenic fungal strains can compromise host immunity (Shofman and Degani 2025b). The current study tested an innovative clay-based method that enables the controlled release of azoxystrobin, providing targeted protection for corn during its vulnerable early growth stages, when LWD pathogen penetration and colonization occur. The substrates used for this purpose are based on clay minerals, which can be applied in their natural form or engineered using organic cations to optimize adsorption, release, and bioactivity (Masini and Abate 2021; Nomicisio et al. 2023). The importance of the results aligns with earlier studies driven by comparable goals.

Previously, for fungal pathogen crop protection, kaolin and bentonite were incorporated into starch–alginate beads to control the release of the fungicide thiram and reduce environmental pollution (Singh et al. 2009). Beads with varying clay contents were characterized via Fourier-transform infrared spectroscopy (FTIR), scanning electron microscopy (SEM), energy dispersive X-ray analysis/spectroscopy (EDAX), thermogravimetry, and swelling studies. Compared with kaolin, the formulations showed high entrapment efficiency and modified thiram release, with bentonite providing a slower, non-Fickian diffusion (anomalous diffusion) profile. Another study that exemplifies such a methodology investigated modified bentonite–alginate nanocomposites that release pesticides—including fungicides—in a controlled, pH‑responsive manner (Yang et al. 2024). This approach enhances the precision of pesticide delivery, potentially reducing environmental exposure and improving efficacy.

The design of clay-based controlled-release systems has been used in other agricultural management challenges, such as improving the effectiveness of pesticides and herbicides. For example, in a recent greenhouse study (Shaltiel-Harpaz et al. 2023), a clay-based biopesticide effectively controlled pests in chives without damaging the foliage. This environmentally friendly method involves essential rosemary oil absorbed onto sepiolite, a mineral known for its ability to bind uncharged molecules (Aranda et al. 2018; Shuali et al. 2011). Another work demonstrated that clay–surfactant formulations can be developed to enable the slow release of herbicides (Galán-Jiménez et al. 2013). In this study, in vitro and soil tests confirmed delayed release and improved bioactivity compared with those of commercial formulations.

It is reasonable that the effectiveness of the treatments only started to appear at the maturation stage when the disease symptoms first appeared. Yet, there were differences in the results of the growth room experiments at the sprouting phase that were related to the clay-AS application method. Seed coating with clay–AS formulations under pathogen stress slightly reduced shoot biomass (12–15%, ns), yet sepiolite–AS suppressed root infection by 92%, whereas bentonite was ineffective, increasing infection by 76%. More inquiring, clay alone (no fungicide) caused a > 600% infection rise over the infected control. It is possible that coating the seeds with clay without fungicide disrupted water uptake and created microenvironments favorable for *M. maydis*, which facilitated pathogen penetration and colonization during the sprouts’ early development.

The results from the growth room and net house trials were consistent at the end of the sprouting phase (days 40 and 42, respectively), regarding the growth indexes. Yet, the pathogen DNA levels in plant roots were relatively high and unaffected by the treatments in the growth room, while drastically reduced by the net house clay-AS treatments. The observed discrepancy in pathogen DNA suppression between the growth room and net house may be attributed to differences in environmental conditions, plant development, and soil–microbe interactions. Net house conditions, being closer to the field, potentially improve plant vigor (including robust root systems that improve fungicide uptake) and immune response, and more favorable slow-release dynamics of the clay–azoxystrobin formulations, collectively enhancing pathogen suppression. In contrast, the uniform and artificial growth room environment may inadvertently favor the pathogen’s early development or reduce the activity of certain protective mechanisms (the seedlings’ microbiome antagonists, plant defense activation), resulting in negligible DNA reduction. Moreover, in the growth room, constant soil moisture or lack of natural drying periods can limit fungicide diffusion or persistence.

To support that, the protective effect of the clay-AS treatments was maintained throughout the season, as indicated by the assessment done at the harvest (growth day 78), demonstrating a superiority of over clay alone. While no AS irrigation (the commercial standard) treatment was included in the net house trial, relevant interpretation can be drawn from a comparable semi-field experiment conducted at nearby farm during the summer of 2022 (Gordani et al. 2023). In that earlier study, the application of Amistar S.C. (5 mL per 10 L pot), applied three times during the season (15, 30, and 44 days after sowing, DAS), resulted in evident improvements in shoot and cob fresh weight (59% and 51%, respectively), along with reductions in dehydration (25%) and pathogen root infection (54%) relative to the infected control, assessed at 78 DAS. While these outcomes were comparable to the current clay–AS treatment for certain parameters (e.g., shoot fresh weight), they were generally inferior to the effects observed for the clay-AS formulations reported here.

Interestingly, the sepiolite–AS treatment provided stronger disease suppression and growth promotion at mid-season, whereas the bentonite–AS combination resulted in the highest plant growth and health parameters toward the end of the growing period. Although this study did not include direct chemical quantification of azoxystrobin in soil (e.g., HPLC or LC–MS), this temporal divergence may reflect differences in fungicide–clay interactions. Additionally, differences in how each clay interacts with soil moisture, microbial activity, and root exudates could influence the timing and duration of fungicide effectiveness. These aspects need to be explored more in dedicated future studies.

Surprisingly, in the net house, bentonite alone had a beneficial effect on both growth and pathogen suppression (see Fig. 5A-C, E and 6C). This treatment was even better than bentonite-AS application at mid-season (day 42). However, such a difference was observed only in cob weight at maturation (day 78). Notably, the disease suppression and growth promotion observed for bentonite alone are consistent with our recent full-season semi-field study on *Macrophomina phaseolina*, in which bentonite application without fungicide significantly increased biomass and reduced pathogen DNA levels (Degani et al. 2026). This indicates a reproducible but context-dependent protective effect, whose mechanism remains unresolved. One possible explanation is that the azoxystrobin dose combined with bentonite may have been phytotoxic at early growth stages—an assumption that requires further investigation. Previous studies have shown that bentonite may have antifungal activity. For example, bentonite loaded with zinc oxide nanoparticles exhibited antifungal activity in laboratory tests against *Aspergillus niger* (de Lucas-Gil et al. 2020). Additionally, this clay might have provided a physical barrier limiting fungal penetration, as shown for kaolin in previous studies (Lamb et al. 2002). Alternatively, bentonite may improve soil moisture retention and structure, promoting beneficial microbial communities known to suppress pathogens such as *Alternaria*, *Bipolaris*, *Fusarium*, *Leptosphaeria*, and *Microdochium* (Chen et al. 2023). Its use in conjunction with copper compounds has also proven effective against fungal diseases such as downy mildew and *Monilia* (Al-Taey et al. 2023). Furthermore, clays such as bentonite are known to bind fungal toxins (e.g., aflatoxins), reducing their harmful impact (Nadziakiewicza et al. 2019).

It is important to recognize the limitations of pot trials in replicating field conditions. The clay application rates reflect the pot-based, proof-of-concept nature of this study and should not be interpreted as field-ready recommendations. Studies report that microbial strains demonstrating strong efficacy in vitro or in greenhouse experiments often fail to translate into effective field performance—an issue largely attributed to the complexity of soil–plant–microbe interactions, formulation challenges, and different impacts under natural conditions (Bizjak-Johansson et al. 2025; Degani et al. 2025c; Ptaszek et al. 2023). Azoxystrobin-based treatments may have stronger effects under greater disease pressure, which is often difficult to replicate in pot experiments, even in a net house.

Currently, azoxystrobin-based treatments for late wilt in corn (Degani et al. 2014, 2018, 2019b, 2020; Matos et al. 2025) are relatively expensive and less practical in fields irrigated by methods such as pivot irrigation. Moreover, pesticide exposure poses significant human health concerns (El Afandi and Irfan 2024), and the extensive use of pesticides in agriculture may affect a diverse range of nontarget species, which is linked to global biodiversity loss (Wan et al. 2025). To mitigate the environmental impact of pesticides, clay-based substrates are being explored as carriers that can prevent pesticide leaching, degradation, or loss of efficacy (Nir et al. 2013; Rytwo et al. 2005, 2008; Rytwo and Rabinowitz 2012). By utilizing adsorption processes and specific molecular interactions, these substrates could enable potentially resource-efficient, long-lasting applications across diverse field conditions while minimizing activity loss due to fungicide movement in the soil.

Moreover, this formulation could significantly reduce overall pesticide usage (by approximately 80% or more compared with drip irrigation) through localized (spot) application and seed coating. The use of azoxystrobin also offers compatibility with biological control strategies. A combination of azoxystrobin and Trichoderma or bacteria was recently demonstrated as an efficient strategy for managing late wilt (Gordani et al. 2023; Matos et al. 2025). Previous studies identified high-potential *Trichoderma* species for *M. maydis* biocontrol applications (Degani and Dor 2021; Degani et al. 2021a; Elshahawy and El-Sayed 2018). Thus, combining biological and chemical management practices to protect plants against phytopathogens should be considered, particularly when single active molecule-based fungicides are used (Ons et al. 2020).

There is a significant risk of evolving azoxystrobin-resistant pathogen strains, particularly when this fungicide is used extensively over consecutive growing seasons (Castroagudín et al. 2015; Fernández-Ortuño et al. 2010a; Leadbeater 2015). Fungicides with a single-site mode of action, such as azoxystrobin, are especially prone to resistance development (Massi et al. 2021). Azoxystrobin, a member of the strobilurin class, acts as a quinone outside inhibitor (QoI) that disrupts mitochondrial respiration by binding to the quinol oxidation (Qo) site of the cytochrome bc1 complex in the fungal electron transport chain, thereby blocking ATP synthesis (Fernández-Ortuño et al. 2010b). Resistance to QoI fungicides has already been reported in more than 20 fungal genera, including *Rhizoctonia solani*, *Alternaria alternata*, *Botrytis cinerea*, *Venturia inaequalis*, and *Mycosphaerella graminicola* (Koehler et al. 2019). Although, to our knowledge, there is no data on azoxystrobin resistance mutations in *M. maydis*, the potential for resistance development is evident. Therefore, it is essential to implement fungicide resistance management strategies (Koehler et al. 2019). Exploring alternative chemical agents for integration into the *M. maydis* management program may expand our options for preventing the emergence of resistance. In addition, the long-term effectiveness of fungicides can be enhanced by combining active ingredients with different modes of action (Brent and Hollomon 1995; Davies et al. 2021).

Bridging the gap between this clay-based experimental stage and its application in agriculture will require substantial further work. The following efforts should focus on field-scale validation of the new clay-azoxystrobin preparation, formulation refinement, combination with biological control agents, and optimization of its application method. By using the methodology demonstrated here, similar clay‒fungicide combinations could be developed and adjusted to protect crop plants from additional soil‒borne pathogens.

## Conclusions

Corn late wilt disease (LWD), induced by the phytopathogenic fungus *Magnaporthiopsis maydis*, exhibits endemic prevalence in Israel, Egypt, Spain, Portugal, India, and several other nations, yet remains relatively obscure on a global scale. The ramifications of LWD in high-risk areas are profound, prompting the continuous pursuit of scientific endeavors to devise effective control strategies. Presently, the primary management method is based on cultivating corn varieties exhibiting resistance to the pathogen. However, this approach faces challenges, as aggressive fungal strains can potentially overcome these resistant cultivars’ immunity. This study demonstrates the potential of clay–azoxystrobin (clay–AS) formulations as a novel and targeted strategy for managing late wilt disease (LWD) in corn. Both sepiolite- and bentonite-based carriers enabled controlled fungicide release, with sepiolite-AS providing stronger early- to mid-season protection and bentonite-AS maintaining higher efficacy in later growth stages. The results highlight the critical influence of carrier properties, environmental conditions, and application method on treatment performance. Direct chemical quantification of azoxystrobin release or persistence in soil (e.g., HPLC or LC–MS–based residue analysis) was beyond the scope of the present study and should be addressed in future field-oriented investigations. Importantly, bentonite alone showed unexpected mid-season benefits, suggesting that certain clays may confer intrinsic antifungal or plant growth–promoting effects. While the clay–AS approach offers advantages in fungicide localization, reduced application frequency, and compatibility with biological control agents, the risk of resistance development to azoxystrobin—common to single-site fungicides—necessitates its integration into diversified disease management programs. Future research should focus on field-scale validation, optimization of formulation and application methods, and evaluation of combinations with biological control organisms. Expanding this approach to other active ingredients and target pathogens could broaden its agricultural utility while reducing environmental impacts associated with conventional pesticide use.

## Supplementary information

Below is the link to the electronic supplementary material.

Supplementary Material 1

## Data Availability

All the data generated or analyzed during this study are included in this published article and its supplementary materials.
