# Supplementary material for: New azoxystrobin clay carrier to control corn late wilt disease
Source: World J Microbiol Biotechnol. 2026 Mar 14;42(3):137. doi: 10.1007/s11274-026-04876-3 (PMC12988907; doi:10.1007/s11274-026-04876-3)
Supplement: Supplementary file 1 — Supplementary Material 1 [file 11274_2026_4876_MOESM1_ESM.docx]

**Supplementary** **Figure S1**. Growth and pathogen infection parameters in the 2023 growth room pot experiment, assessed 20 days after sowing. Treatments consisted of azoxystrobin (Amistar S.C.; Syngenta, Basel, Switzerland) adsorbed onto sepiolite and bentonite clays (yellow and blue bars, respectively), applied directly to the sowing pit at sowing. (**A**) shoot fresh weight, (**B**) phenological development (leaf count), (**C**) shoot height, and (**D**) relative quantity of *M. maydis* (*Mm*) DNA in root tissues, normalized to plant cytochrome c oxidase (*Cox*) gene levels. Controls: Healthy (green bars) or uninf. – non-inoculated; Infected or DDW inf. (black bars) – inoculated without AS-based treatments; AS irrigation (gray bars) – Amistar S.C. (1.25 mL commercial product) applied via irrigation on days 0, 9, and 18 after sowing. Abbreviations: Sep – sepiolite; Bent – bentonite; AS – azoxystrobin; DDW – clay mixed with deionized distilled water (no fungicide). Bars represent the mean of 4–9 biological replicates; error bars indicate standard error. Different letters (a–d) above bars indicate statistically significant differences between treatments (*p* < 0.05), based on the Kruskal–Wallis test.

**Supplementary** **Figure S2**. Growth and pathogen infection parameters in the 2023 growth room pot experiment, assessed 20 days after sowing. Treatments consisted of azoxystrobin (Amistar S.C.; Syngenta, Basel, Switzerland) adsorbed onto sepiolite and bentonite clays (yellow and blue bars, respectively), applied as seed coating (SC). (**A**) shoot fresh weight, (**B**) phenological development (leaf count), (**C**) shoot height, and (**D**) relative quantity of *M. maydis* (*Mm*) DNA in root tissues, normalized to plant cytochrome c oxidase (*Cox*) gene levels. Controls: Healthy (green bars) or uninf. – non-inoculated; Infected or DDW inf. (black bars) – inoculated without AS-based treatments; AS irrigation (gray bars) – Amistar S.C. (1.25 mL commercial product) applied via irrigation on days 0, 9, and 18 after sowing. Abbreviations: Sep – sepiolite; Bent – bentonite; AS – azoxystrobin; DDW – clay mixed with deionized distilled water (no fungicide). Bars represent the mean of 5–9 biological replicates; error bars indicate standard error. Different letters (a–d) above bars indicate statistically significant differences between treatments (*p* < 0.05), based on the Kruskal–Wallis test.

**Supplementary** **Table S1**. Semi field peeking on day 7. ^1^

| **Controls** | | **SEP** | | | | **BENT** | | | |
| --- | --- | --- | --- | --- | --- | --- | --- | --- | --- |
| Healthy | Infected | AS uninf. | AS inf. | DDW uninf. | DDW Inf. | AS uninf. | AS inf. | DDW uninf. | DDW Inf. |
| 205%^a^ | 100%^c^ | 210%^a^ | 190%^ab^ | 210%^a^ | 171%^bc^ | 205%^a^ | 129%^c^ | 214%^a^ | 195%^ab^ |

^1^ Effect of clay–azoxystrobin formulations on late wilt disease in corn grown in the net house, assessed 7 days after sowing. Treatments included azoxystrobin adsorbed onto sepiolite or bentonite clays (highlighted in yellow and blue, respectively), applied directly to the sowing pit at sowing. Controls: Healthy (highlighted green) or uninf. – non-inoculated; Infected (highlighted in gray) or DDW inf. – inoculated without AS-based treatments. Abbreviations: Sep – sepiolite; Bent – bentonite; AS – azoxystrobin; DDW – clay mixed with deionized distilled water (no fungicide). Data represent the mean ± standard error of 9 biological replicates. Different letters (a–c) indicate statistically significant differences between treatments (*p* < 0.05), based on the Kruskal–Wallis test.
